# Enhancing Frontal Sinus Surgery: Assessing the Long-Term Impact of Free Grafts and Flaps in Draf III Procedures

**DOI:** 10.3390/jpm14040396

**Published:** 2024-04-09

**Authors:** Argyro Leventi, Vasileios Chatzinakis, Georgia Evangelia Papargyriou, Christos Georgalas

**Affiliations:** 1 Endoscopic Skull Base Centre Athens, Hygeia Hospital, 151 23 Athens, Greece; aleventi@hygeia.gr (A.L.); vchatzinakis@hygeia.gr (V.C.); gpapargyriou@hygeia.gr (G.E.P.); 2 Medical School, University of Nicosia, Nicosia 2408, Cyprus

**Keywords:** Draf III, free grafts, flaps, restenosis, neo-ostium, retrospective

## Abstract

The frontal sinus medial drainage —Draf Type III (modified endoscopic Lothrop) procedure, has become a cornerstone in frontal sinus surgery over the last three decades. Despite its widespread acceptance, challenges such as restenosis and neo-ostium closure persist, prompting the exploration of various preventive techniques. In this retrospective study, we analyzed data from 111 patients who underwent the Draf III procedure between November 2015 and November 2023, with a mean follow-up period of 3 years and 11 months. Approximately two-thirds of patients (64%) had undergone previous sinus surgery and 16% a previous Draf III. Over half of the patients had inflammatory conditions, with the majority being chronic rhinosinusitis with nasal polyps (CRSwNP) (46%), while 15% were diagnosed with malignant sinonasal tumors, and 23% with benign sinonasal tumors, of which the commonest was osteoma, accounting for 14 cases. The mean follow-up period was 3 years and 11 months. We focused on evaluating the efficacy of mucosal flaps and free grafts in preventing neo-ostium closure. Although it appears that there is no statistically significant correlation between flap usage and the need for revision surgery or ostium patency maintenance overall, subgroup analysis highlighted the benefits of flap reconstruction in patients with chronic rhinosinusitis with nasal polyps. In this subgroup, the use of flaps or grafts reduced the rate of neo-ostium stenosis from 20% to 0% (*p* < 0.05). Overall revision rate was 11.7%—however this was 8% in patients without acute inflammation at the time of surgery and went up to 31% in the presence of pus in the frontal recess (*p* = 0.02). This study contributes to the existing literature by providing insights into long-term outcomes, the enduring effectiveness of interventions in frontal sinus surgery, and especially the importance of taking into account the underlying pathology when assessing long-term outcomes.

## 1. Introduction

In 1991, Professor Wolfgang Draf introduced the endoscopic medial frontal sinus drainage procedure, commonly referred to as the Draf type III procedure but also known as endoscopic modified Lothrop (EMLP), bilateral frontal sinus drill-out, or nasofrontal approach IV [1]. Over the past three decades, this frontal sinus procedure has gained broad acceptance, both as an independent surgical intervention and as a vital component of comprehensive treatment strategies for a range of pathologies. These conditions encompass chronic rhinosinusitis (CRS), mucoceles, and both benign (such as osteomas, inverted papillomas, ossifying fibromas, etc.) and malignant tumors affecting the frontal sinus. Additionally, it extends to cases involving cerebrospinal fluid (CSF) leaks and patients requiring transcribriform or transfrontal approaches for accessing anterior skull-base tumors via endonasal routes [2].

Despite its success in numerous cases, challenges such as restenosis, scarring, and neo-ostium closure persist, often resulting in less-than-optimal outcomes and necessitating revision surgery. A number of techniques to prevent neo-ostium stenosis have been introduced [3,4,5,6], ranging from different types of random or vascularized flaps to free grafts [7,8,9,10,11,12,13]; however, there have been only a handful of studies assessing their effectiveness. Most of these studies, although well designed, have rather small number of participants or other limitations, such as short follow-up periods or a different case mix of patients and pathologies. Consequently, there is a need for comprehensive research to evaluate the outcomes of these procedures thoroughly.

In this context, we present a retrospective study aimed at conducting an analysis of patient outcomes and surgical data sourced from our institution. Through this investigation, we seek to contribute to the existing body of knowledge surrounding frontal sinus surgery, with a particular focus on evaluating the impact of free grafts and flaps in the Draf III procedure, as well as factors contributing to restenosis.

## 2. Materials and Methods

This study employed a retrospective study design, including all patients who underwent the Draf III procedure at the Department of Endoscopic Paranasal and Skull Base Surgery of Hygeia Hospital in Athens between November 2015 and November 2023. All surgeries were performed by the senior author (C.G.).

Patients eligible for the Draf III procedure presented with chronic rhinosinusitis (CRS) and its associated complications, acute rhinosinusitis (ARS), mucoceles, benign and malignant sinonasal tumors (SNT), and cerebrospinal fluid (CSF) leaks. Additionally, the procedure was performed as part of a broader surgical intervention necessitating frontal sinus management. Patients with incomplete medical records were excluded.

The Draf III procedure was performed under general anesthesia. Both lateral-to-medial and medial-to-lateral surgical techniques were adopted, according to previously described methods [1,2,8,14]. Throughout the procedure, a 30-degree endoscope was predominantly used, with occasional utilization of a 70-degree endoscope for lateral visualization. Furthermore, surgical precision was enhanced by the routine application of a guided navigation system.

Two vascularized flaps – namely septoturbinal flap and lateral-based nasoseptal flap, as described by Khoueir et al. and Fiorini et al. [9,15], as well as free mucosal grafts, as described by Illing et al. [16] and Wang et al. [8], were harvested at the outset of each procedure and meticulously maintained in an intact state throughout the process. Nasal packing was not utilized.

Follow-up appointments typically commenced one to two weeks postoperatively, with a follow up visit to remove the silicon sheet at 4 to 6 weeks and subsequent visits scheduled based on individual pathologies and healing progress. These follow-up sessions commonly occurred at two to three months post-surgery and then at six months and one year after surgery. Most patients (and virtually all the patients with CRS and malignant tumors) were followed up for a minimum of five years, as we believe that neo-ostium stenosis does not only occur between 12 and 24 months, as reported [17,18], but also in subsequent years [3]. During these visits, the status of the neo-ostium was assessed endoscopically to determine whether it remained open (defined as a patency allowing for adequate sinus drainage) or had closed (defined as occlusion hindering sinus drainage). Computed tomography (CT) or magnetic resonance imaging (MRI) scans were conducted solely based on clinical indications, rather than serving as a means to assess the neo-ostium. Additionally, all patients received nasal irrigation, while nasal corticosteroids were administered to those diagnosed with chronic rhinosinusitis.

Data analysis was conducted using the Statistical Package for the Social Sciences (SPSS) version 29. Descriptive statistics, encompassing means, standard deviations, and frequencies, summarized the demographic and clinical characteristics of the study participants. Inferential statistics, such as Pearson’s chi-square tests or Fisher’s exact tests as required, were employed to explore associations between variables. Furthermore, multivariate analysis techniques, including regression analysis, were utilized to identify significant predictors of neo-ostium closure. The significance level for all statistical analyses was set at *p* < 0.05.

## 3. Results

Between November 2015 and November 2023, 111 patients underwent the Draf III procedure, either independently or as part of a combined procedure. The patient cohort comprised 46 females and 65 males, with a mean age of 49 years (range: 13–78 years). Notably, over half of the procedures addressed inflammatory conditions, with the majority being chronic rhinosinusitis with nasal polyps (CRSwNP) (46%), while 15% were for malignant and 23% for benign sinonasal tumors (Table 1). The most common benign tumor encountered was osteoma, accounting for 14 cases. The mean follow-up period was 3 years and 11 months.

Among the patients, almost two thirds (64%) had undergone previous sinus surgery, while 16% had undergone a previous Draf III procedure. Overall, 32% had a history of asthma, 23% were smokers, and 28% tested positive on skin prick tests (SPT) for allergies. 

Interestingly, 14% exhibited evidence of acute inflammation, characterized by pus in the frontal sinus, at the time of Draf III procedure. These were patients who underwent surgery after undergoing multiple courses of culture-driven antibiotics preoperatively and were often patients with ARS complications, such as Potts puffy tumor.

In our analysis, we included both patients who underwent primary Draf III and those who underwent revision Draf III procedures from other centers. The overall revision rate was 11.7%, with 13 out of 111 patients requiring revision surgery due to obstruction of the neo-ostium. The majority of patients requiring revision (10 out of 13) had undergone Draf III for CRSwNP or its complications, while the remaining 3 for benign sinonasal tumors—2 with inverted papillomas and 1 with a cholesterol granuloma of the frontal sinus. None of the patients undergoing Draf III for malignant tumors or CSF leak required revision surgery for ostium stenosis (Table 1). The revision rate for the group of patients who underwent primary Draf III was 11%, while for the subgroup who underwent revision Draf III, it was 17%. However, this difference did not appear to be statistically significant.

**Scheme 1:**
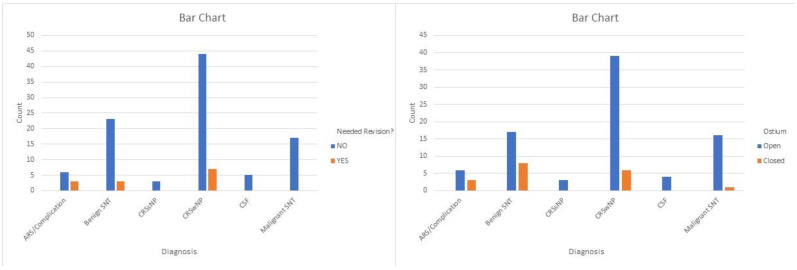
Revision necessity vs. ostium status variation among various pathologies. ARS, acute rhinosinusitis; CRS, chronic rhinosinusitis; SNT, sinonasal tumors; CRSsNP, chronic rhinosinusitis without nasal polyps; CRSwNP, chronic rhinosinusitis with nasal polyps; CSF, cerebrospinal fluid.

Similarly, there was a very significant difference in rates of re-stenosis according to the pathology—ranging from 0% for patients with sinonasal malignant tumors to 20% for patients undergoing Draf III for CRSwNP (Scheme 1).

Upon examining the subgroup of patients who underwent Draf III for chronic rhinosinusitis with nasal polyps (CRSwNP), notable trends emerged. The incidence of asthma, allergies, aspirin hypersensitivity, and elevated eosinophils in both blood and polyp specimens was significantly higher in this subgroup. More specifically, 62% of the patients undergoing Draf III for CRSwNP suffered from asthma, 53% had a documented allergy, 21% had increased eosinophils in the blood, 50% had increased eosinophils in the polyps, and 52% had positive skin prick tests. The prevalence of allergy (*p* < 0.005), asthma (*p* < 0.001), and positive skin prick tests (*p* = 0.005) was higher in patients with CRSwNP; however, this was not the case for eosinophilia (*p* = 0.09) or aspirin hypersensitivity (*p* = 0.17) compared to the other subgroups. The incidence of smokers within this subgroup remained unchanged. 

**Scheme 2:**
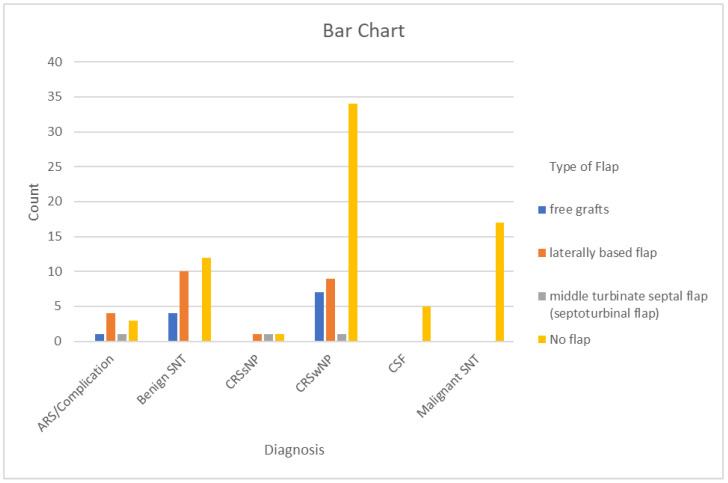
Different types of flaps/grafts across diagnosis variants. ARS, acute rhinosinusitis; CRS, chronic rhinosinusitis; SNT, sinonasal tumors; CRSsNP, chronic rhinosinusitis without nasal polyps; CRSwNP, chronic rhinosinusitis with nasal polyps; CSF, cerebrospinal fluid.

**Figure 1:**
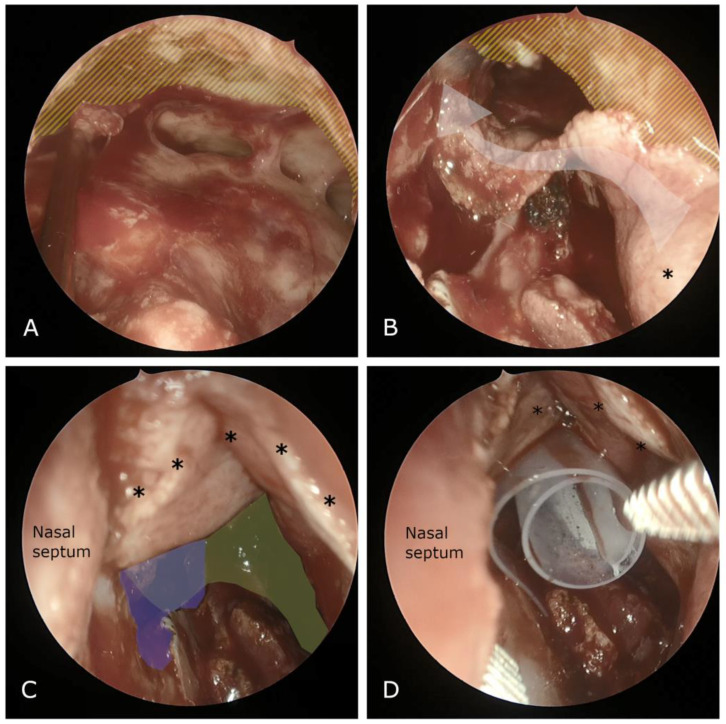
(**A**) Intraoperative perspective depicting the unified frontal sinuses subsequent to a Draf III procedure, prior to flap placement. Note the extensive bone exposure following the drill-out; (**B**) the laterally based flap (left side), mobilized for coverage of the exposed bone (asterisk denotes base of the flap); (**C**) conclusive view displaying the flap in situ (asterisks denote flap placement, green indicates neo-ostium, purple represents septal window); and (**D**) a 0.25 mm silastic sheet utilized to secure the flap (asterisks) postoperatively for 4–6 weeks.

In approximately two-thirds of the patients we used the lateral-to-medial (inside-out) approach, while in oncological cases involving benign and malignant sinonasal tumors we used the medial-to-lateral approach. Mucosal flaps and free grafts were utilized in 36% of cases, with the exception of malignant cases where they were never employed. Specifically, free grafts were utilized in 30.8% of cases with flaps/grafts, laterally based flaps in 61.5%, and middle turbinate septal flaps in 7.7% (Scheme 2, Figure 1).

We assessed whether diagnosis, allergy, skin prick tests (SPTs), eosinophils in the blood or polyps, smoking, asthma, surgical technique, the use of flaps, and the presence of acute inflammation during surgery were statistically associated with Draf III closure. Only acute inflammation at the time of surgery was significantly associated (*p* = 0.02, Fisher exact test) with the need for revision surgery. Specifically, nearly one-third of patients who underwent surgery with concurrent acute inflammation, particularly within the frontal sinus, required subsequent Draf III revision surgery. In contrast, the rate of revision surgery was 8% in the group of patients without acute inflammation at the time of surgery. In total, out of 13 patients requiring revision surgery, 5 were patients undergoing Draf III for acute inflammation (out of a total of 16 such patients).

Interestingly, we also observed that within the same subgroup of patients, the utilization of the lateral-to-medial technique was correlated with higher rates of neo-ostium patency, as demonstrated by the statistical analysis (*p* = 0.007).

While analyzing the entire group of 111 patients, we found no significant correlation between flap usage and the need for revision surgery or ostium patency maintenance. However, the diverse range of diagnoses and pathologies should be considered, potentially introducing bias. Flap usage was avoided in malignant tumors, where neo-ostium stenosis is less common, while we used it more frequently in inflammatory pathologies like CRSwNP, where the rate of restenosis is higher.

**Scheme 3:**
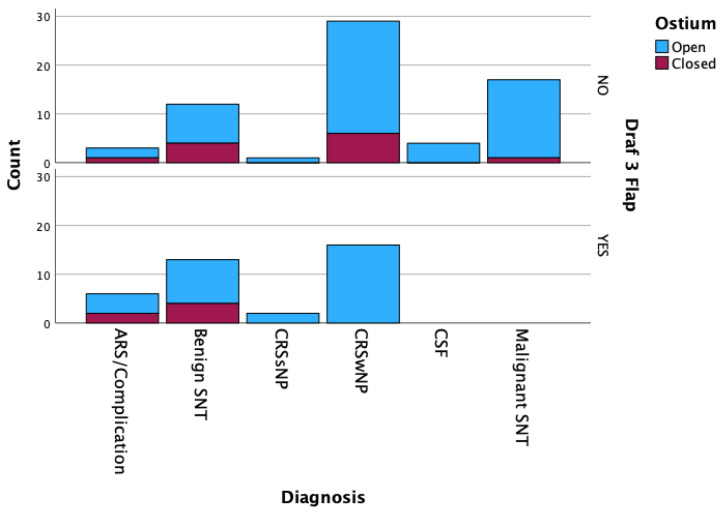
Ostium status by diagnosis and flap/graft usage in Draf III Procedure (graphical representation).

Therefore, we attempted to compare outcomes among patients according to pathology. Our analysis suggests that in patients with CRSwNP, the use of flaps/grafts may offer a borderline advantage. Among the 16 patients who underwent Draf III for CRSwNP and received flap/graft reconstruction, none developed ostium closure, whereas 6 out of 29 patients who did not undergo flap reconstruction experienced ostium closure (*p* < 0.05) (Scheme 3, Table 2).

## 4. Discussion

While the Draf III procedure has gained wide acceptance since its introduction in 1991, challenges persist in accurately predicting and effectively preventing neo-ostium stenosis. As already described, the process of bone drilling, along with the resultant exposure of bone, has been recognized as a predisposing factor for complications such as osteitis, scarring, osteogenesis, and an increased risk of neo-ostium closure [9,19].

In this context, numerous authors have explored various factors associated with closure and suggested diverse surgical approaches to prevent it [20]. For instance, Kang ST et al. have advocated for the repeated use of expandable polyvinyl acetate (EPA) packing to prevent stenosis of the frontal sinus ostium [21]. AlQahtani et al. proposed a double-vascularized nasoseptal flap to prevent restenosis after a Draf III procedure, as part of a laboratory investigation [11]. Furthermore, in recent years, alongside surgical techniques, there has been an emergence of sinus implants designed to locally deliver steroids to the sinus mucosa. These implants hold promise for reducing inflammation and promoting healing following surgery. However, despite their potential benefits, additional studies are required to assess their efficacy specifically in the context of frontal sinus surgery [22,23].

In our study, our objective was to assess the effectiveness of mucosal flaps and free grafts in our patient cohort, particularly in relation to neo-ostium outcomes. A few authors have pursued similar studies in recent years, publishing patient series with varying sample sizes and follow-up durations. Bastianelli et al. examined healing outcomes using anterior pedicled flaps in frontal sinus drill-out procedures in 12 patients. They found no significant difference between patients with mucosal flaps and those without; however, long-term data were limited due to healthcare regulations related to COVID-19 [7].

Ye et al. investigated the outcomes of mucosal flaps in 43 patients who underwent Draf IIb and Draf III procedures, with a follow-up period of 1 year. They observed a significant reduction in neo-ostium restenosis [10]. However, they included both Draf II and Draf III procedures, which could introduce selection bias in their results and make conclusions less applicable to Draf III procedures.

Wang et al. conducted a study involving 50 patients who underwent Draf III procedures with or without grafts or flaps. They measured frontal neo-ostium dimensions at 6 weeks, 6 months, and 12 months postoperatively, concluding that restenosis was reduced in the group that received grafts or flaps [8]; however, the clinical significance of the increased size of the ostium, per se, as opposed to revision surgery or complete stenosis is unclear. 

Illing et al. conducted a study on graft outcomes in a cohort of 67 patients, with a mean follow-up period of 34 months, during which they observed excellent outcomes [16] in their patient population, which, however, encompassed a large proportion of patients with non-inflammatory conditions (CSF leaks and tumors).

Fischer et al. did the biggest study and examined data from 123 patients over a follow-up period of up to 24 months. Their analysis revealed that the use of a lateral pedicle flap in reconstructing mucosal defects during frontal sinus surgery enhances the likelihood of sustained drainage [24]. However, their average follow-up was only 2 years and 7 months, excluding, in this way, patients who may undergo stenosis later. Indeed, in our series, at least one-third of stenosis cases happened after the first two years of follow-up, especially in patients with CRSwNP, and followed the resurgence of their underlying disease.

In our analysis, we included a cohort of 111 patients, making this study one of the largest single-center investigations and with a mean follow-up period of 47 months the one with the largest follow-up. Our overall revision rate was 11.7 percent; however, this varied significantly according to pathology—from zero in malignant tumors to almost 20% in inflammatory disease. We found no significant correlation between free grafts and mucosal vascularized flap usage and the necessity for revision surgery or the maintenance of ostium patency overall. 

However, it is crucial to consider the diverse range of diagnoses and pathologies within our patient cohort. Draf III could be part of many different procedures regarding the treatment of inflammatory diseases such as chronic rhinosinusitis (CRS) with or without nasal polyps, benign or malignant sinonasal tumors (SNT), cerebrospinal fluid (CSF) leak repair, etc. [2]. What is important when performing comparisons between studies and techniques is the “elephant in the room”, which is the underlying pathology. Performing a Draf III for a non-inflammatory (skull base or oncological) pathology is very different compared to CRSwNP. This heterogeneous population may introduce bias, as flaps are typically not employed in patients undergoing oncological procedures for malignancies, who are less likely to develop neo-ostium stenosis anyway. Conversely, flaps are commonly utilized in patients with inflammatory pathologies, such as refractory chronic rhinosinusitis with nasal polyps (CRSwNP), who demonstrate significantly higher rates of restenosis according to our data. In this subgroup, we found that the use of free grafts and mucosal vascularized flaps significantly prevented neo-ostium closure. This suggests that the application of these techniques may offer advantages in mitigating the risk of restenosis, particularly in patients with inflammatory conditions such as CRSwNP.

The fact that the persistence of inflammation leads to a high rate of neo-ostium closure was also observed in cases of acute inflammation with the presence of pus in the frontal sinus during surgery, regardless of the underlying pathology. This suggests a strategy of avoiding frontal sinus surgery during acute inflammatory episodes, choosing to provide long-term intravenous antibiotics and perform the procedure when the acute inflammation has resolved. We do understand, however, that this may represent a conundrum, as sometimes, and despite culture-driven antibiotics, the inflammation may not resolve and may be associated with life-threatening complications. We have shown in the past that in such cases, drainage of the frontal sinus may provide an advantage in terms of reducing complications, albeit at the risk of long-term ostium stenosis [25].

Regarding factors such as asthma, allergies, aspirin hypersensitivity, and elevated eosinophils in both blood and polyps among patients with CRSwNP, we did not observe any significant associations, consistent with the findings of Schlosser et al. in their series of 44 patients [26]. However, studies by Tran et al. [5] and Fischer et al. [24] did not confirm this in their patient cohorts.

**Figure 2:**
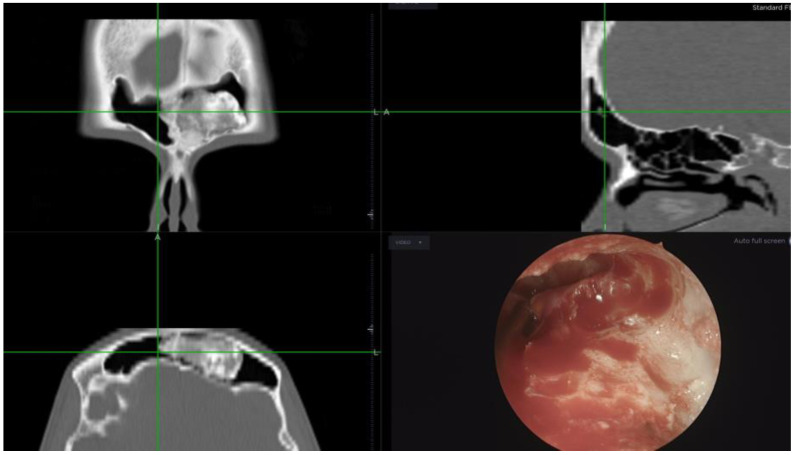
Intraoperative navigation image captured during a Draf III procedure aimed at frontal osteoma removal. Observe the extensive drilling necessary to access the lateral aspect of the lesion.

**Figure 3:**
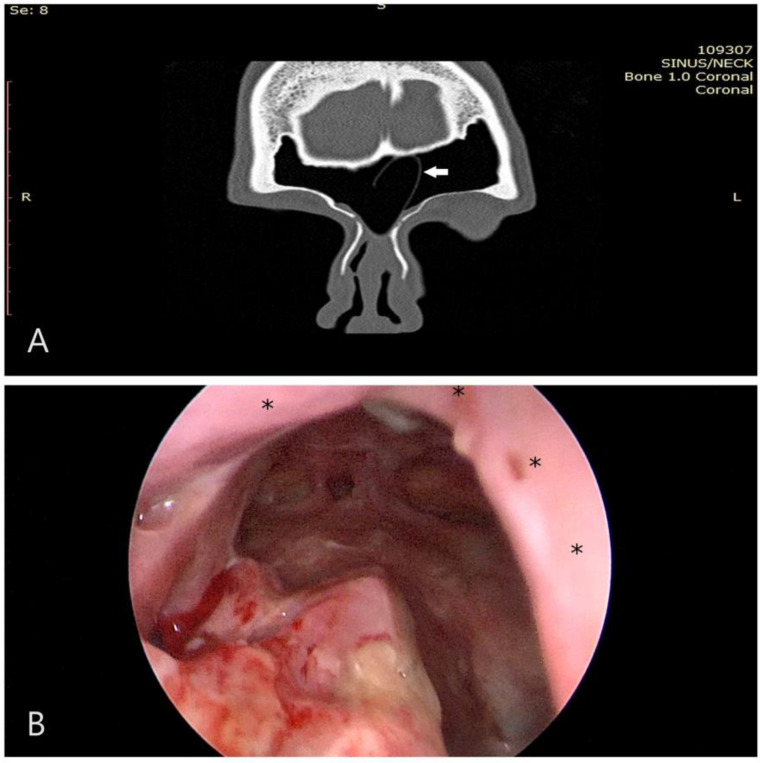
(**A**) CT scan of the same case, taken 3 weeks postoperatively. Observe the presence of the silastic sheet (indicated by the white arrow); (**B**) subsequent follow-up image, captured 6 weeks postoperatively. The neo-ostium is prominently open, and the flap is thoroughly integrated (asterisks).

Another notable finding we observed was the high rate of neo-ostium closure in benign SNT. The vast majority of benign SNT cases in our cohort were osteomas—fourteen osteomas located in the frontal sinus, necessitating extensive drilling during surgery. It is well established, as noted by Rajapaksa et al. [19], that excessive drilling can contribute to restenosis (Figure 2 and Figure 3). Consequently, we hypothesize that this excessive drilling may account for the observed stenosis in this particular subgroup. The use of concurrent flaps and drug-eluding devices may offer a way to reduce neo-ostium stenosis in the future in such patients, and we are currently investigating this.

Furthermore, it is essential to acknowledge the inherent limitations of our retrospective study design. The lack of randomization in allocating patients to groups with or without flaps or grafts may introduce biases into the analysis, potentially impacting the interpretation of the study’s outcomes. Therefore, while our study offers valuable real-world insights, future research should prioritize prospective randomized controlled trials to validate these findings rigorously.

Finally, it is worth highlighting that this study represents one of the most extensive examinations conducted thus far, particularly in regard to the duration of follow-up, which extends over 3 years and 11 months. This prolonged observation period not only enriches the depth of our findings but also offers valuable insights into the enduring effectiveness of the interventions under investigation. As previously elucidated, our findings highlight the elevated rates of neo-ostium restenosis observed in inflammatory conditions such as chronic rhinosinusitis with nasal polyps (CRSwNP) over prolonged follow-up periods. By adhering to specific selection criteria for identifying patients suitable for biologic therapies, there is potential to alter the disease course [27,28,29], including the status of the neo-ostium, accordingly. We expect that the advent of biologic treatments will play a role in improving the efficacy of Draf III surgery in patients with CRSwNP.

## 5. Conclusions

In conclusion, our retrospective analysis of 111 patients undergoing the Draf III procedure provides valuable insights into the effectiveness of mucosal flaps and free grafts in preventing neo-ostium closure, and it identifies factors influencing restenosis. It is crucial to note that the underlying pathology significantly impacts surgical outcomes, particularly in non-inflammatory conditions compared to chronic rhinosinusitis with nasal polyps. While no significant correlation was found between flaps/grafts usage and the need for revision surgery or ostium patency maintenance overall, subgroup analysis suggests that the use of flaps and grafts does provide benefits for patients with chronic rhinosinusitis with nasal polyps. Acute inflammation during surgery emerged as a significant predictor of revision surgery. These findings provide important insights into the long-term effectiveness of interventions in frontal sinus surgery and how they could complement additional treatment with biologic therapies such as monoclonal antibodies.

## Figures and Tables

**Table 1:** Diagnoses among study participants and the actual breakdown of revision surgery by diagnosis.

		Needed Revision?		Total
		No	Yes	
Diagnosis [n(%)]	ARS/Complication [9(8.1%)]	6 (66.7%)	3 (33.3%)	9 (100%)
	Benign SNT [26(23.4%)]	23 (88.5%)	3 (11.5%)	26 (100%)
	CRSsNP [3(2.7%)]	3 (100%)	0 (0%)	3 (100%)
	CRSwNP [51(45.9%)]	44 (86.3%)	7 (13.7%)	51 (100%)
	CSF [5(4.5%)]	5 (100%)	0 (0%)	5 (100%)
	Malignant SNT [17(15.3%)]	17 (100%)	0 (0%)	17 (100%)
Total		98 (88.3%)	13 (11.7%)	111 (100%)

ARS, acute rhinosinusitis; CRS, chronic rhinosinusitis; SNT, sinonasal tumors; CRSsNP, chronic rhinosinusitis without nasal polyps; CRSwNP, chronic rhinosinusitis with nasal polyps; CSF, cerebrospinal fluid.

**Table 2:** Ostium Status by diagnosis and flap/graft usage in Draf III Procedure (tabular representation).

Diagnosis			Draf III Flap/Graft	
			No	Yes
ARS/Complication off CRS	Ostium	Open	66.7%	66.7%
		Closed	33.3%	33.3%
Benign SNT	Ostium	Open	66.7%	69.2%
		Closed	33.3%	30.8%
CRSsNP	Ostium	Open	100.0%	100.0%
		Closed	0%	0%
CRSwNP	Ostium	Open	79.3%	100.0%
		Closed	20.7%	0.0%
CSF	Ostium	Open	100.0%	0%
		Closed	0%	0%
Malignant SNT	Ostium	Open	94.1%	0%
		Closed	5.9%	0%
Total	Ostium	Open	81.8%	83.8%
		Closed	18.2%	16.2%

ARS, acute rhinosinusitis; CRS, chronic rhinosinusitis; SNT, sinonasal tumors; CRSsNP, chronic rhinosinusitis without nasal polyps; CRSwNP, chronic rhinosinusitis with nasal polyps; CSF, cerebrospinal fluid.

## Data Availability

Detailed data are unavailable due to privacy restrictions (date of operation and diagnosis).
